# The Effect of HiPIMS Pulse Conditions on the Microstructural, Mechanical, and Tribological Properties of TiB_2_ Coatings on Steel Substrates

**DOI:** 10.3390/ma18204699

**Published:** 2025-10-13

**Authors:** Daniel Kottfer, Karol Kyzioł, Mária Kaňuchová, Marta Kianicová, Michal Žitňan, Ewa Durda, Marianna Trebuňová, Dávid Medveď, Patrik Kľučiar

**Affiliations:** 1 Faculty of Special Technology, Alexander Dubček University of Trenčín, Ku Kyselke 469, 911 06 Trenčín, Slovakia; marta.kianicova@tnuni.sk (M.K.); patrik.kluciar@tnuni.sk (P.K.); 2 Faculty of Materials Science and Ceramics, AGH University of Krakow, A. Mickiewicza Av. 30, 30 059 Kraków, Poland; edurda@agh.edu.pl; 3 Institute of Mountainous Sciences and Environmental Protection, Faculty of Mining, Ecology, Process Control and Geotechnology, Technical University of Košice, Park Komenského 19, 043 84 Košice, Slovakia; maria.kanuchova@tuke.sk; 4 Centre for Functional and Surface Functionalized Glass, Alexander Dubček University of Trenčín, Študentská 2, 911 50 Trenčín, Slovakia; michal.zitnan@tnuni.sk; 5 Department of Biomedical Engineering and Measurement, Institute of Special Engineering Processes, Faculty of Mechanical Engineering, Technical University of Košice, Letná 9, 042 00 Košice, Slovakia; marianna.trebunova@tuke.sk; 6 Institute of Materials Research, Slovak Academy of Sciences, Watsonova 47, 040 01 Košice, Slovakia; dmedved@saske.sk

**Keywords:** surface engineering, HiPIMS, TiB_2_ coatings, mechanical properties, tribology

## Abstract

This study examines the impact of varying pulse conditions on the properties of titanium diboride (TiB_2_) coatings deposited by high-power impulse magnetron sputtering (HiPIMS). The coatings were prepared on steel substrates using an industrial-scale system. During the experiments, the HiPIMS frequency and pulse width were systematically varied to examine their influence on the coating’s microstructural, mechanical, and tribological properties. The obtained results show a correlation between process parameters and coating performance. A maximum hardness of 39.7 GPa and a coefficient of friction (CoF) as low as 0.68 were achieved. The best combination of mechanical properties was observed for coatings prepared in a frequency range of 600–1000 Hz and with a pulse width of 50 µs. Notably, the optimal tribological properties and surface roughness were obtained at 800 Hz and a 50 µs pulse width. This work demonstrates that fine-tuning HiPIMS pulse conditions is crucial for achieving high-quality TiB_2_ coatings with enhanced functional performance.

## 1. Introduction

Plasma-based deposition techniques of interest for improving the surface properties of engineering components. While titanium (Ti) coatings are valuable as adhesive interlayers due to their thermal expansion properties, their low hardness (ca. 2.5 GPa) and poor wear resistance make them unsuitable for many demanding applications [1,2,3,4,5,6,7]. To overcome these limitations, the addition of boron during deposition creates titanium diboride (TiB_2_), a high-performance ceramic coating. With its hexagonal crystal structure, high melting point (3225 °C), and exceptional hardness (over 30 GPa), TiB_2_ is a prospective material for increasing wear resistance and extending the service life of industrial parts [8]. TiB_2_ coatings can be deposited by chemical vapor deposition (CVD) [9,10], physical vapor deposition (PVD) including pulsed laser deposition (PLD) [11], magnetron sputtering (MS) [12,13,14,15,16,17,18,19,20,21,22,23,24,25,26], and high-power impulse magnetron sputtering (HiPIMS) [20,27,28,29,30,31,32,33,34,35,36,37,38,39,40], and has emerged as a promising technology. Compared to conventional magnetron sputtering (MS), HiPIMS generates a high plasma density, which results in a denser microstructure and superior mechanical properties [20,27,28,29,30,31,32,33,34,35,36,37,38,39,40]. However, a notable drawback is the significantly lower deposition rate, which presents a challenge for large-scale industrial use [36]. The existing body of research on TiB_2_ coatings has primarily focused on two areas: investigating the impact of technological parameters and doping the coatings with specific elements [24,25,26,32,33,34,35]. For instance, studies have explored the effects of bias voltage [27], deposition temperature [30,31], and their combination in the structure of elements like Al, Cu, Si, and N [23,24,25,32,33,35,39,40]. While these studies have provided valuable insights, they have largely confined their parameter variations to narrow intervals. For example, Nedfors et al. [28] and Zhang et al. [27] examined frequency and bias voltage changes, but their research work did not provide a comprehensive view of how a wide range of these parameters influences the coating’s properties.

In contrast to existing works, this study addresses a critical gap in the literature by providing a systematic and expanded investigation into the effects of a broad range of HiPIMS pulse conditions on the properties of TiB_2_ coatings. The novel advancement of this work is its comprehensive analysis, which investigates how the simultaneous, wide-ranging variation in HiPIMS pulse parameters—pulse frequency (from 600 Hz to 4000 Hz) at a constant pulse width (50 µs) and pulse width (from 50 µs to 200 µs) at a constant frequency (800 Hz)—influences the growth rate, mechanical, tribological, and structural properties of TiB_2_ coatings. A key aspect of this work is the use of an industrial-scale deposition system, which ensures that the results are directly applicable to practical manufacturing processes [7,28,34]. The obtained results were compared with the literature to advance the scientific understanding of HiPIMS deposition of high-performance TiB_2_ coatings.

## 2. Experimental Details

### 2.1. Coating Deposition

**Figure 1:**
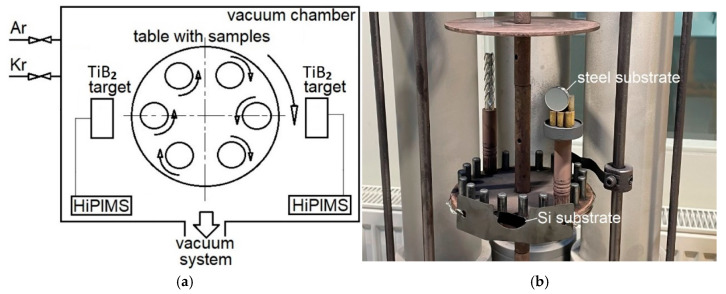
(**a**) Scheme of coating system CC800/9 CemeCon AG used during deposition process (Commercservice, s.r.o. Prešov, Slovakia); (**b**) sample view in the vacuum chamber.

The study on the deposition of TiB_2_ coatings was divided into two main parts. First, a set of coatings was deposited at a constant pulse width of 50 µs, while the frequency was systematically varied across a wide range of 600, 800, 1000, 2000, and 4000 Hz. In the second part, a frequency of 800 Hz was selected to investigate the effect of pulse width, with values ranging from 50, 70, 100, and 150 to 200 µs. All experiments were performed using a CemeCon AG CC800/9 industrial coating system (Figure 1), with the technological parameters selected from the full range permitted by the equipment.

For chemical composition analysis by X-ray photoelectron spectroscopy (XPS), Si(001) substrates (15 × 10 × 0.5 mm) were used. Additionally, steel substrates (diameter ca. 22 mm, thickness ca. 4 mm) were prepared for pin-on-disk tribological tests and to analyze mechanical behavior. The chemical composition of the steel, according to STN EN 19 830, was (at.%) 0.8 C, 0.45 Mn, 0.45 Si, 4.15 Cr, 6.60 W, 4.95 Mo, and 1.9 V. Both Si (001) and steel samples were ultrasonically cleaned in acetone for 12 min before being placed in the vacuum chamber (Figure 1b) at a distance of 60 mm to 130 mm from the targets.

After the chamber was evacuated to a base pressure of 4 × 10^−3^ Pa, the substrates were cleaned via ion etching. This pre-treatment was performed for 30 min using a gas mixture of Ar (99.999% purity) at 200 cm^3^/min and Kr (99.999% purity) at 50 cm^3^/min.

The etching parameters included a pulse length of 1.6 µs and a frequency of 240 kHz. During this stage of surface modification, the samples were simultaneously heated to a temperature of 300 °C. Following the pre-treatment, the pressure was gradually increased to 0.8 Pa.

The TiB_2_ coatings were deposited over a period of 3 h and 55 min using a working gas flow of Ar at 570 cm^3^/min. Sputtering was performed from two TiB_2_ targets (99.5% purity, Ti/B stoichiometric ratio = 1:2), each powered by an HiPIMS magnetron at 4.5 kW. The process parameters were a voltage of 550 V, a current of 21 A, and a bias voltage of −60 V. The samples underwent planetary motion at a rotational speed of 2 rpm, with a target-to-substrate distance of 60 mm and a current density of 3.0 mA/cm^2^.

### 2.2. Coating Characterization

The microstructure of the samples was evaluated by scanning electron microscopy (SEM) using Apreo2 and Scios2 DualBeam microscopes (ThermoFisher Scientific, Brno, Czech Republic). The thickness and grain morphology were determined from cross-sectional views of the coated samples at magnifications ranging from 20,000× to 100,000×.

Crystalline phases and crystallite size were analyzed using X-ray diffraction (XRD) on a PANalytical Empyrean diffractometer (Empyrean Almelo, The Netherlands) with Cu Kα radiation (λ = 1.5405 Å, 45 kV, 40 mA). Diffraction patterns were collected at a scan rate of 1.9°/min and analyzed using High Score Plus software (v.5.1.0, PAN Analytical) with the PDF-2 database (2022) [41]. The crystallite size was calculated using the Scherrer method [42]. Surface roughness (S_a_) was measured with an OLYMPUS LEXT OLS5100-SAF confocal microscope (Olympus Europa SE and Co. KG, Hamburg, Germany) over two distinct areas: 32 µm × 32 µm and 258 µm × 258 µm. The chemical composition of the TiB_2_ coatings was determined by X-ray photoelectron spectroscopy (XPS) using a SPECS instrument (Berlin, Germany) equipped with a Phoibos 100 SCD (Berlin, Germany) and a non-monochromatic X-ray source. Surface spectra were recorded at a pass energy of 70 eV, while core spectra were acquired at 30 eV. All spectra were obtained at a base pressure of 1 × 10^−8^ mbar with MgKα excitation (10 kV, 200 W). Data analysis was performed with SpecSLAB2 CASAXPS software (Casa Software Ltd., Teignmouth, UK), using both Shirley and Tougaard baselines for fitting.

Coating adhesion was assessed using the Mercedes test in accordance with the VDI 3198 standard. Indentations were made with a diamond Rockwell indenter (120° apex angle) and evaluated using an OLYMPUS—MX51 optical microscope (Nagano, Japan).

Hardness (H) and Young’s modulus (E) were measured (evaluated as average values from 10 measurements) via indentation on a Bruker Hysitron TI 950 TriboIndenter (Materials Research Laboratory, Urbana, IL, USA) using a diamond Berkovich indenter (E = 1140 GPa, ν = 0.07). Up to ten indentations were performed on each sample. A sinus loading mode at a frequency of 45 Hz was used, with an indentation depth limit of 500 nm to ensure the indenter tip did not penetrate more than 1/10th of the coating’s total thickness. The hardness and Young’s modulus were calculated from the measured indentation depth within the range of 100 nm to 200 nm, and average values were reported.

The coefficient of friction (CoF) and wear behavior were evaluated using a pin-on-disk test on a Bruker TriboLab UMT (Bruker Austria GmbH, Wien, Austria) with a linear track. A hardened steel ball (G40, 1.4034 DIN 5401), with a diameter of 4.76 mm and a hardness of HRC 52-60 (5.5–7.0 GPa), was used as the counterpart. The test parameters were path length of 10 mm, speed of 10 mm/s, test duration of 7200 s, and a normal load of 5 N. Wear was quantified by measuring the cross-sectional area of the wear track using the OLYMPUS LEXT OLS5100-SAF confocal microscope. The cross-sectional area was measured at the halfway point of the wear track after the test was completed.

## 3. Results and Discussion

### 3.1. Thickness, Microstructure, Shape and Grain Size

**Figure 2:**
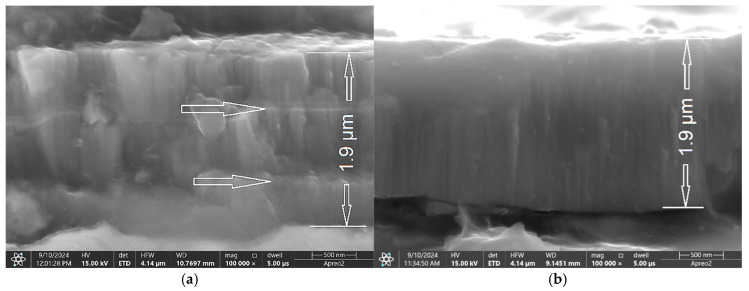

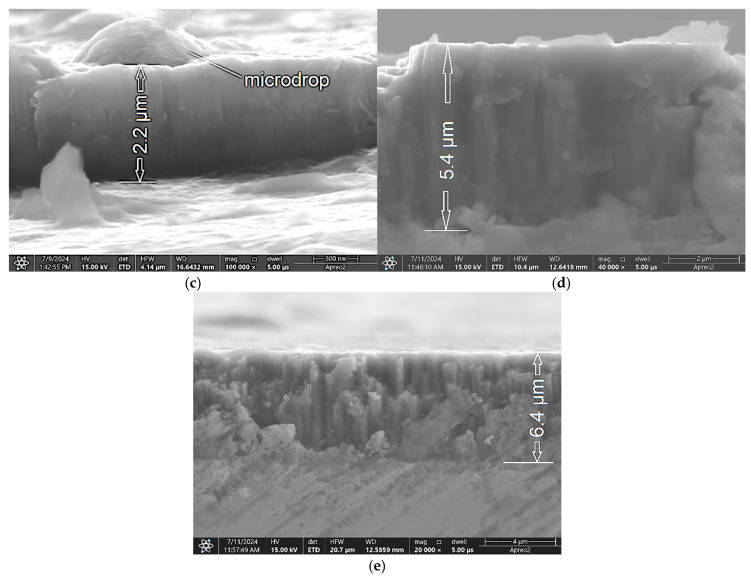
Cross-sectional view of TiB_2_ coatings deposited on steel at a pulse width of 50 µs and various frequencies: (**a**) 600 Hz; (**b**) 800 Hz; (**c**) 1000 Hz; (**d**) 2000 Hz and (**e**) 4000 Hz.

The thickness of the obtained TiB_2_ coatings (Figure 2a–c) increased with both frequency and pulse width. When the frequency was increased from 600 to 1000 Hz (with a pulse width of 50 µs), the coating thickness increased ca. 15%, from 1.9 µm up to 2.2 µm.

**Figure 3:**
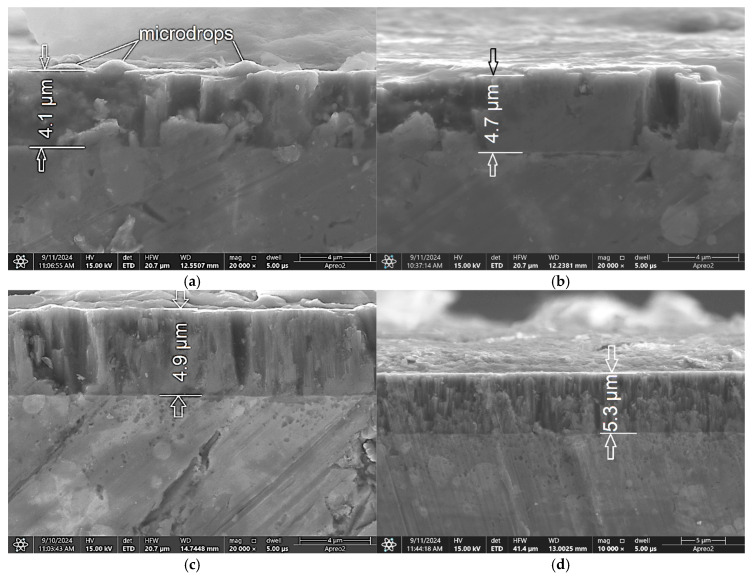
Cross-sectional view of TiB_2_ coatings deposited on steel at a frequency of 800 Hz and pulse widths of (**a**) 70 µs; (**b**) 100 µs; (**c**) 150 µs and (**d**) 200 µs.

A further increase in frequency to 4000 Hz resulted in a more significant increase of ca. 300%, from 2.2 µm to 6.4 µm (Figure 2c,e). For the coating deposited at 600 Hz and a 50 µs pulse width, two short interruptions in deposition are visible (Figure 2a, see arrows). Similarly, the pulse width also impacted the coating thickness. As the pulse width increased, the thickness of the coating also increased, from 1.9 µm (at 50 µs; Figure 2b) to 5.3 µm (at 200 µs; Figure 3d). The overall effect of both frequency and pulse width on coating thickness is similar, though the increase is more significant when frequency is varied (Figure S1a, Supplementary Materials). The coating structure is columnar with integrated microdroplets (Figure 3a–d). The microstructure consists of columnar grains, as described by the Thorntons diagram for a T/Tm ratio of 0.093 (T = 300 °C) and a pressure of 30 mTorr [43]. The obtained coating is significantly denser without nanogaps. Grains with a cauliflower-like structure are also integrated into the coating (Figure 2c). Nedfors et al. [28] deposited a TiB_2_ coating on an Al_2_O_3_ substrate and found that the thickness decreased with increasing frequency. This is consistent with our results (Figure S1a). Their obtained thickness was 1.76 µm (growth rate of 22 nm/min. over an 80 min deposition time).

When scaled to a 235 min deposition time, the thickness would be 5.2 µm, which is in good agreement with the thickness measured in conducted experiments (Figure 3d). In contrast, Sala et al. [34] deposited a TiB_2_ coating on a Si substrate, achieving a thickness of 3.03 µm. This is 30% less than the 4.1 µm thickness measured in this study (Figure 3a and Figure S1b).

**Figure 4:**
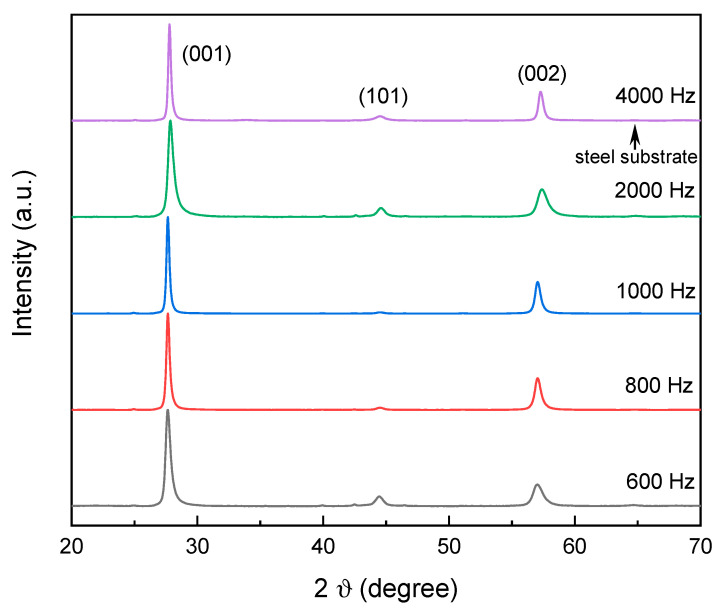
X-ray diffraction θ-2θ scans of the series of coatings sputtered with increasing pulse frequency.

In the next stage, the samples of the TiB_2_ coating, composed of granules and crystals, was characterized via X-ray diffraction (XRD) to determine crystallographic orientation and grain size. The XRD patterns of coatings deposited on steel substrates at varying frequencies are presented in Figure 4.

Analysis of the XRD spectra reveals three main reflections at 27.8°, 44.6°, and 57.4°, which correspond to the TiB_2_ phase (PDF 00-035-0741) and confirm the formation of a hexagonal h-TiB_2_ structure.

The peaks corresponding to the (001) and (002) planes, at 27.8° and 57.4°, respectively, exhibit significantly higher relative intensities. In contrast, the peak for the (101) orientation at 44.6° shows very low intensity, while the (100) and (110) diffraction peaks were not observed. This strong preferred grain orientation for TiB_2_ is consistent with findings reported in previous studies [34,44,45]. Other minor phases were also detected: a low-intensity peak at 35.3° belonged to the (201) plane of TiB (PDF 01-073-2148), and a peak at 40.1° can be attributed to the (101) plane of Ti (PDF 00-005-0700).

In turn, the analysis of the (001) peak revealed a full width at half maximum (FWHM) ranging from 0.30° to 0.60°, with no clear dependence on the pulse frequency. However, the peak’s position shifted to higher 2θ values as the pulse frequency increased. This shift indicates a compression of the c-lattice parameter, decreasing from 3.223 Å at 600 Hz to 3.207 Å at 4000 Hz. For further analysis, the crystal sizes were calculated from the (001) peak using the Scherrer method [42]. The results, presented in Table S1 (Supplementary Materials), show that crystal sizes range from 14 up to 28 nm as the pulse frequency increases from 600 Hz to 4000 Hz, with no clear correlation to frequency.

**Figure 5:**
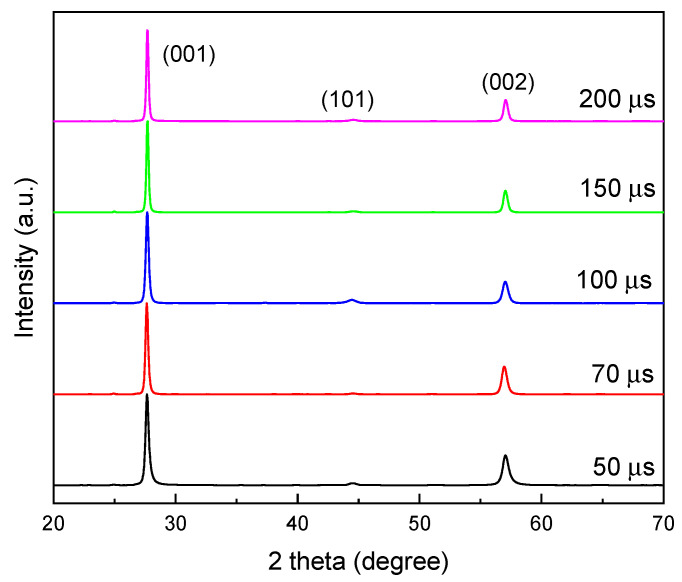
X-ray diffraction θ-2θ scans of the series of coatings sputtered with increasing pulse width.

On the other hand, Figure 5 presents the XRD θ-2θ diffractograms of the TiB_2_ coatings deposited using different pulse widths.

All observed peaks can be definitively assigned to the TiB_2_ phase, and a strong (001) crystallographic texture is clearly visible in all analyzed samples. No significant shift was observed in the position of the (001) peak as the pulse width was varied. Similarly, the values for the c-lattice parameter, which range from 3.220 Å at 200 µs to 3.223 Å at 50 µs, do not show a significant trend. The full width at half maximum (FWHM) of the TiB_2_ (001) diffraction peak ranges from 0.23° to 0.36°, and the calculated grain size for pulse widths from 50 to 200 µs is in the range of 23 to 35 nm (Table S2, Supplementary Materials).

**Figure 6:**
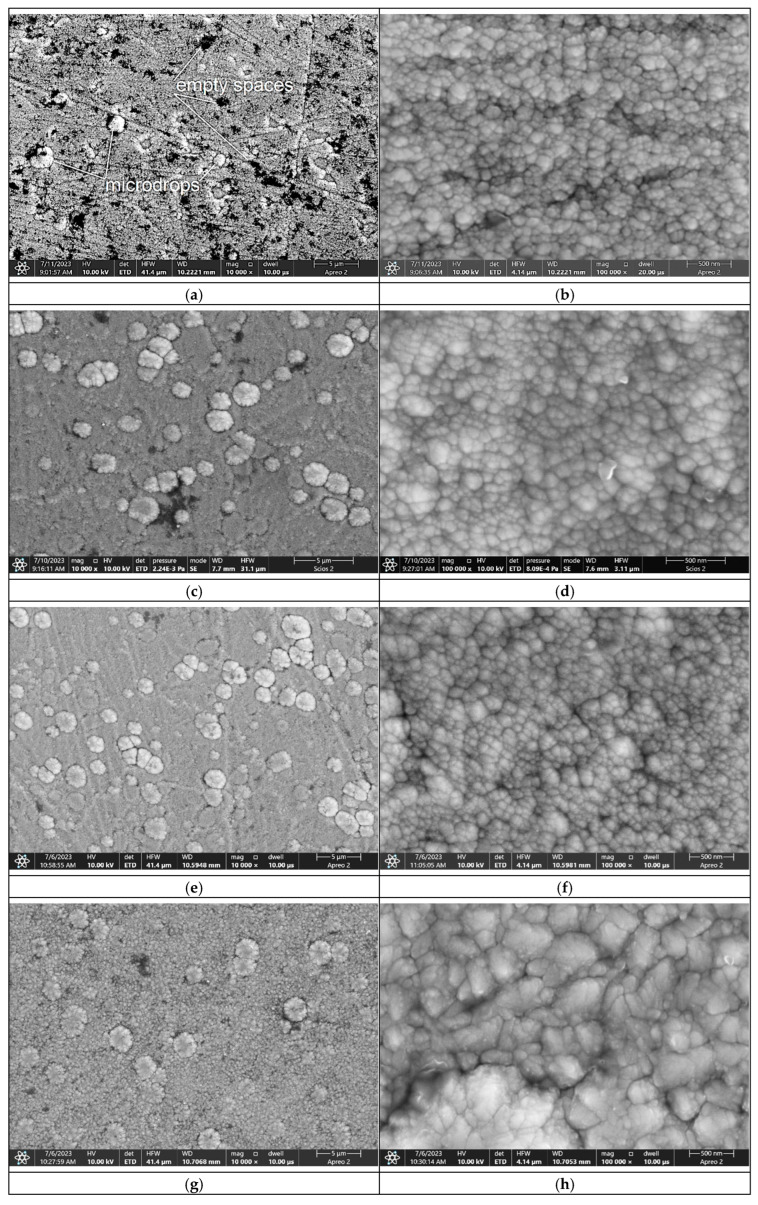

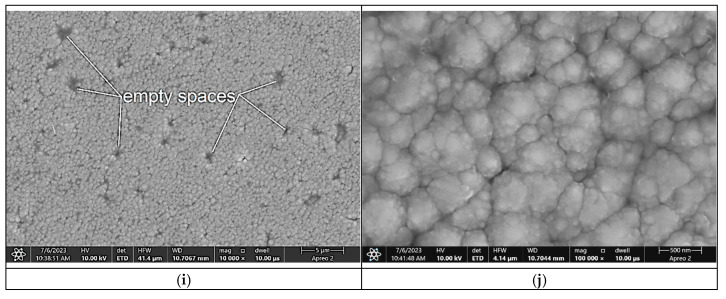
SEM image of the TiB_2_ coating surface deposited on steel at a constant pulse width of 50 µs and various frequencies: (**a**,**b**) 600 Hz; (**c**,**d**) 800 Hz; (**e**,**f**) 1000 Hz; (**g**,**h**) 2000 Hz; (**i**,**j**) 4000 Hz.

The obtained research results for grain size show a notable difference from some previous studies. Sala et al. [34] measured a crystal size of only 8 nm for a TiB_2_ coating deposited at a frequency of 800 Hz, a pulse width of 70 µs, and a magnetron power of 4.5 kW. This result is 20 nm smaller than the crystal sizes obtained in our study, a difference that was reflected in their reported values of hardness and Young’s modulus. However, our research results correspond with those of Zhang et al. [27], who achieved a TiB_2_ coating with a crystal size of 24 nm using specific deposition parameters of −50 V bias and a temperature of 300 °C. The grains exhibited a columnar shape with cauliflower-shaped ends. At a deposition frequency of 600 Hz, grain diameters ranged from 30 nm up to 250 nm (Figure 6b,d,f). Additionally, the coating contained larger cauliflower-shaped formations with diameters of ca. 1 µm up to 2 µm and voids measuring roughly 1.0 µm × 1.0 µm to 2.5 µm (Figure 6a).

As the frequency increased to 800 Hz and 1000 Hz, the grain shape and size remained consistent. However, the coating surface showed an increased number of cauliflower-shaped formations, with diameters up to 2 µm (Figure 6c,e). These formations were also visible at a frequency of 2000 Hz (Figure 6g) but were not observed at 4000 Hz (Figure 6i). In contrast, the grain size increased at higher frequencies (Figure 6h,j), reaching values of approximately 50 nm to 500 nm.

As confirmed, the HiPIMS method delivers high current and power density in individual pulses, generating an abundance of ionized particles and a high plasma density [37]. During this process, cathodic arc discharge, or arcing, can occur, characterized by a sudden current increase and a target voltage drop [27,38,46]. This arcing ejects microscopic target residues, leading to unwanted droplets and defects in the deposited coatings [27,38,46]. The embedded droplets observed in the TiB_2_ coatings (Figure 6a,c,e,g) are caused by this arcing phenomenon. Our experiments show that arcing produces embedded droplets in TiB_2_ coatings at frequencies ranging from 800 Hz to 2000 Hz (with a pulse width of 50 µs). At a frequency of 600 Hz, only small, partially embedded droplets and significant voids are visible. Conversely, at 4000 Hz (with a pulse width of 50 µs), these droplets are not formed (Figure 6i,j). SEM images of the TiB_2_ coating at 100,000× magnification are shown in Figure 6b,d,f,h,j.

**Figure 7:**
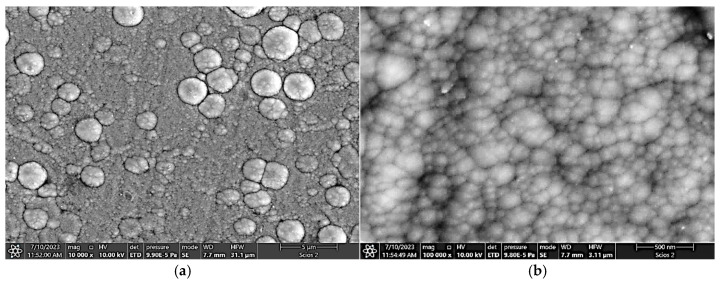

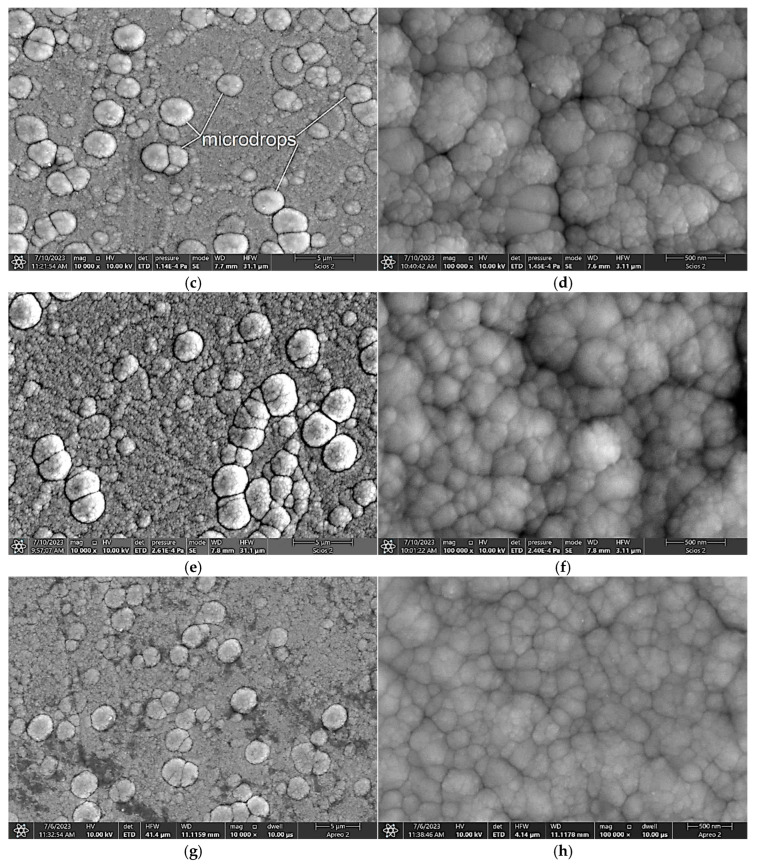
SEM image of the TiB_2_ coating surface deposited on steel at a constant frequency of 800 Hz and various pulse widths: (**a**,**b**) 70 µs; (**c**,**d**) 100 µs; (**e**,**f**) 150 µs and (**g**,**h**) 200 µs.

In the case of a constant frequency of 800 Hz with increasing pulse width, the coating at 50 µs and 70 µs pulse widths (Figure 6a and Figure 7a) consists of grains with cauliflower-shaped formations, each approximately 1 to 2 µm in diameter. The individual grains themselves range from 30 nm to 250 nm in diameter (Figure 6b and Figure 7b) and the presence of voids is similar for these two pulse widths.

As the pulse width increases, the number of voids significantly decreases (Figure 7d,f). At a pulse width of 200 µs, the TiB_2_ coating has a dense cauliflower structure with no visible voids (Figure 7h). Importantly, the embedded microdroplets, caused by arcing, begin to appear in the TiB_2_ coating as the pulse width increases beyond 60 µs (Figure 7c,e,g). In contrast, at a 50 µs pulse width, only microdroplets that are not embedded in the coating are present (Figure 7a). This is likely because the coating’s thickness is only 1.9 µm, which is very small compared to the microdroplets’ diameter of 500 nm to 2 µm (Figure 6b). The energy of a pulse is a direct function of the source power, frequency, and pulse width. With a constant power source and a constant pulse width, a lower frequency leads to higher pulse energy. Therefore, an increase in frequency should theoretically lead to more intense microdroplet formation. Similarly, a shorter pulse width results in higher pulse energy. This implies that the maximum amount of microdroplets should be produced at a maximum power, minimum pulse width, and minimum frequency. In our experiment, where the power was held constant, the number of microdroplets was expected to increase with higher pulse energy. The impact of frequency on the number and size of microdroplets appears to be relatively less significant than the influence of pulse width. In the case of varying frequency at a constant pulse width, the maximum size of microdroplets was approximately 2 µm (at 1000 Hz and 2000 Hz; Figure 6c,d). Interestingly, no microdroplets appeared at a frequency of 4000 Hz. At 600 Hz, microdroplets up to 1 µm in size were visible (Figure 6a). In contrast, the influence of pulse width was more pronounced. The size of microdroplets reached up to ca. 3 µm (Figure 7). This can be attributed to the significantly higher pulse energy, which causes more sputtering from the target [27,38,46]. Finally, the other research results [7] also confirm that the chemical composition and microstructure were significantly altered by increasing the bias voltage, resulting in the best mechanical and nanowear properties and lowest surface roughness of sputtered TiB_2_ coatings at −200 V.

### 3.2. Hardness, Young’s Modulus, Roughness and Adhesion

**Figure 8:**
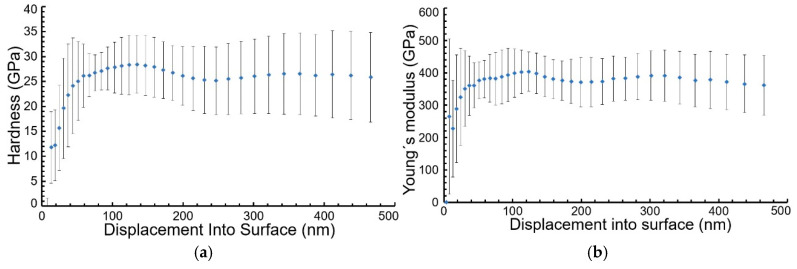
Hardness modulus (**a**) and Young’s modulus (**b**) values of TiB_2_ coating deposited on steel substrate at a frequency of 2000 Hz and pulse width of 50 µs, depending on distance into surface.

The hardness and Young’s modulus of the TiB_2_ coating deposited at a frequency of 2000 Hz and a pulse width of 50 µs are shown in Figure 8a,b, respectively.

**Figure 9:**
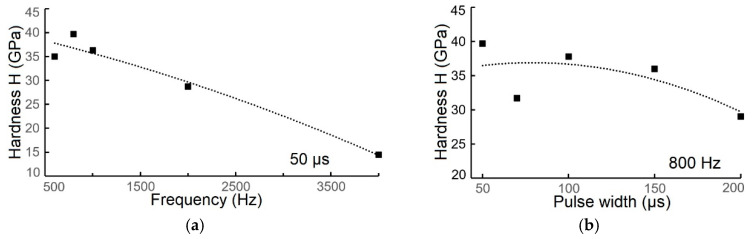

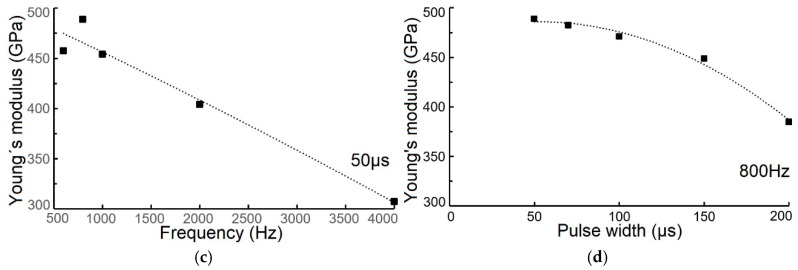
Hardness modulus (**a**,**b**) and Young’s modulus (**c**,**d**) values of TiB_2_ coating deposited on steel substrate at various values of frequency (at pulse width of 50 µs) as well as at various values of pulse width at a frequency of 800 Hz, depending on distance into surface.

When the pulse width was held constant at 50 µs, the hardness of the coatings decreased from a maximum of 39.7 GPa (at 800 Hz) to a minimum of 14.5 GPa (at 4000 Hz); Figure 9a. A similar trend was observed when the frequency was held constant at 800 Hz and the pulse width was increased, with hardness decreasing from 39.7 GPa (at 50 µs) to 29 GPa (at 200 µs), Figure 9b. The observed maximum hardness value was higher than that reported by Zhang et al. (29 GPa at 300 °C, 50 V bias) but lower than their maximum of 44 GPa (at 200 °C, 50 V bias) [27]. The value is comparable to that of Nedfors et al. (45.5 GPa at 500 Hz, −60 V bias; 42 GPa at 1000 Hz, −60 V bias) [28]. Hardness increases with decreasing grain size (Tables S1 and S2—Supplementary Materials).

Furthermore, the measured hardness was ca. 20% higher than the value reported by Sala et al. [34] and about 10% lower than that measured by Polyakov et al. [21]. In the case of Young’s modulus, observed values had a similar trend to hardness. With increasing frequency, it decreased from 489 GPa (at 800 Hz) to 307 GPa (at 4000 Hz), Figure 6c. Similarly, increasing the pulse width led to a decrease in Young’s modulus from 489 GPa (at 800 µs) to 385 GPa (at 4000 µs), Figure 6d. The value we obtained is comparable to those reported by Nedfors et al. (~480 GPa at 600 Hz and 1000 Hz with a −60 V bias) [28] and Zhang et al. [27]. Authors from studies [28,32,34] deposited TiB_2_ coatings using the same C800/9 CemeCon AG device used in our experiment. Sala et al. [34] reported a Young’s modulus of E = 447 ± 9 GPa, which is ca. 10% lower than the value measured in this study.

In conclusion, the hardness of the obtained TiB_2_ coatings decreased significantly with increasing frequency and also decreased with increasing pulse width. This is likely influenced by the coating’s microstructure, and specifically the presence of voids and the possible formation of other phases like Ti and TiB, which have significantly lower hardness values (Ti ≈ 2.5 GPa [1,2]; TiB ≈ 12.0 GPa [5]). The minimum measured hardness of 14 GPa (at 4000 Hz frequency, 50 µs pulse width) can be correlated with a maximum crystal size of 28 nm and the occurrence of gaps in the coating (Figure 6i). The detailed composition of the TiB_2_ coatings at various frequencies and pulse widths, and its impact on mechanical and structural properties, could be a subject for further research. Importantly, Nedfors et al. [28] confirm that lower frequencies provide a higher degree of ionization, which does, however, increase the compressive residual stress in the coatings, resulting in harder coatings.

**Figure 10:**
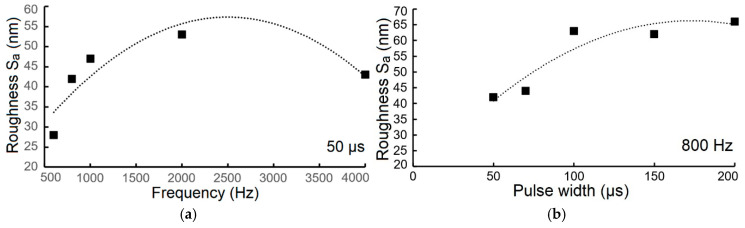
The roughness value (S_a_) of TiB_2_ coatings deposited on steel substrate depending on (**a**) frequency and (**b**) pulse width.

In the case of the surface roughness of the evaluated TiB_2_ coatings, measured as the arithmetic mean deviation (S_a_), it showed a clear dependence on both deposition frequency and pulse width. With a constant pulse width of 50 µs, the coating roughness increased with frequency, from 28 nm to a maximum of 53 nm at 2000 Hz (Figure 10a).

However, at 4000 Hz, the roughness decreased to 43 nm. The presence and characteristics of embedded microdroplets directly influence this roughness, as shown in Figure S2a,c,e,g,i (Supplementary Materials), with their density and size visible in the corresponding figures (Figure S2b,d,f,h,j, Supplementary Materials). As the pulse width was increased, the roughness also increased. At a constant frequency of 2000 Hz, the roughness rose from 42 nm to 66 nm (Figure 10b). Specifically, at 800 Hz, a 200 µs pulse width resulted in a roughness of 66 nm (Figure 7b). In both cases, the roughness was influenced by the integrated microdroplets in the coating (Figure S3a,c,e,g), with their density and size clearly visible in the Figure S3b,d,f,h (Supplementary Materials).

In conclusion, both frequency and pulse width influence the roughness of the TiB_2_ coating. The roughness of the evaluated coating decreases as both technological parameters increase at a frequency range between 800 Hz and 4000 Hz.

Importantly, the surface roughness is directly influenced by the presence of integrated microdroplets within the coating, as evidenced by the microstructural images in Figures S2a,c,e,g,i and S3a,c,e,g (Supplementary Materials). The density and size of these microdroplets, which are crucial determinants of roughness, are clearly visible in the corresponding higher-magnification images (Figures S2b,d,f,h,j and S3b,d,f,h, Supplementary Materials). Based on these analyses, it is concluded that both the deposition frequency and the pulse width significantly affect the surface roughness (S_a_) of the TiB_2_ coating. The data suggest a clear relationship: as both technological parameters are increased within the 800 Hz to 4000 Hz range, the resulting roughness of the coating decreases. This implies that controlling these parameters is essential for achieving a smoother surface.

Importantly, adhesion of the evaluated TiB_2_ coatings using the Mercedes test had a grade of HF1 to HF2 (Figure S4, Supplementary Materials). It can be concluded that the evaluated coatings are suitable for use in engineering applications.

### 3.3. Chemical Composition

The chemical composition of the TiB_x_ coatings was analyzed using X-ray Photoelectron Spectroscopy (XPS). For example, the surface spectrum of a thin coating (600 Hz, 50 µs) confirms the presence of 74.9% Ti (1 s) and 25.10% B (1 s), Figure S5 (Supplementary Materials). The presented values for boron and titanium elements, especially the effect of frequency at a constant pulse width of 50 µs as well as the effect of pulse width at a constant frequency of 800 Hz, were also investigated (Table 1 and Table 2, respectively).

**Figure 11:**
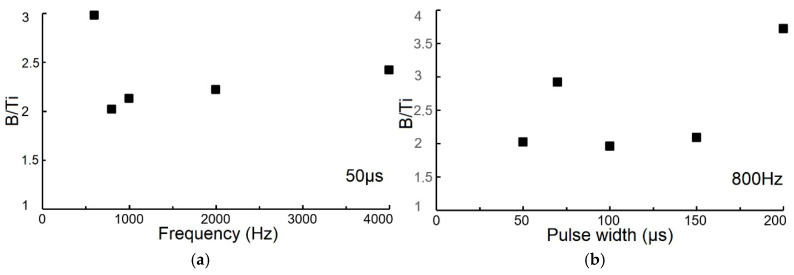
Dependence of the chemical composition (B/Ti) of TiB_2_ coating on (**a**) frequency and (**b**) pulse width.

As the frequency increased (with a constant 50 µs pulse width), the B/Ti ratio increased from 2.0 to 2.4 (Figure 11a). An exception was observed at 600 Hz, where the ratio was 3.0. The stoichiometric and overstoichiometric TiB_x_ coatings obtained in this study are consistent with previous findings [13]. These results are similar to those of Nedfors et al. [28], who deposited TiB_2_ coatings at frequencies from 200 Hz to 500 Hz, obtaining B/Ti ratios from 2.2 to 2.3 and confirming a slight increasing trend. In our experiments, the B/Ti ratio of 3.0 at 600 Hz is significantly higher than the ratio of 2.2 obtained by Sala et al. [34] at 800 Hz and a 70 µs pulse width.

At a constant frequency of 800 Hz, there was a tendency for the B/Ti ratio to increase with increasing pulse width (Figure 11b), which may be a result of the increasing plasma density [7]. The coatings obtained were stoichiometric, except for those deposited at pulse widths of 70 µs and 200 µs. The maximum B/Ti ratio was 3.9 (at 800 Hz, 200 µs), while the lowest value was 2.1 (at 800 Hz, 50 µs). To explain the anomalous B/Ti ratios observed in the data, specifically the high values at 600 Hz (frequency variation) and at 70 µs and 200 µs (pulse width variation), it is important to consider the dynamics of the HiPIMS process.

Our research results correspond to Bakhit et al. [7], who obtained a B/Ti ratio of 1.99 at a 50 µs pulse width, which is in good agreement with our results for the same pulse width. Importantly, the authors demonstrate that stoichiometric TiB_2_ coatings can be obtained by adjusting the length of HiPIMS pulses while maintaining average power and pulse frequency constant, finally operating in power-controlled mode. The slightly increasing trend of the B/Ti ratio with pulse width is consistent with the findings in [7], with the exception of the 70 µs and 200 µs pulse widths (Figure 11b). This increasing tendency is related to a decrease in the negative target ignition voltage due to a limited capacitor bank size. Simultaneously, as the pulse width peak increases, the current density decreases significantly [7].

The anomalous B/Ti ratios can be attributed to several interacting factors inherent to HiPIMS deposition. Firstly, the anomalous ratios may be a consequence of varying degrees of target poisoning caused by residual oxygen and nitrogen in the vacuum chamber. As demonstrated in previous research [28], oxygen has a strong affinity for titanium, which can lead to the formation of titanium oxides (TiO_2_) on the target surface. When the target is poisoned, the sputtering yield of titanium decreases disproportionately compared to boron, which has a higher sputtering rate in the presence of oxygen. This leads to an enrichment of boron in the deposited coating, resulting in an abnormally high B/Ti ratio. The degree of poisoning can be sensitive to subtle fluctuations in process parameters, explaining the observed non-linear behavior. In the other hand, the surface of the TiB_2_ target can become eroded non-uniformly, which affects the sputtering yield of Ti and B atoms [13]. The formation of conical structures, or “coning,” on the target surface due to selective sputtering can alter the effective sputtering area and local plasma density. This can lead to variations in the flux of B and Ti species reaching the substrate, causing the observed fluctuations in the B/Ti ratio. The efficiency of ionization for Ti and B atoms can vary with changes in frequency and pulse width [28,34]. At certain combinations of parameters (such as the 600 Hz frequency and 70/200 µs pulse widths), the plasma conditions might favor the preferential ionization and transport of boron atoms over titanium atoms. This effect would lead to a higher flux of boron ions to the substrate, resulting in a boron-rich coating composition. The high ionization rate of HiPIMS, which is generally beneficial for coating density, can also amplify these compositional discrepancies if not precisely controlled.

### 3.4. Coefficient of Friction and Wear

The wear resistance of TiB_2_ coatings depends mainly on the hardness and microstructure. A microstructure with a grain size of several nanometers (nanostructure), located closely together or without gaps, significantly increases the wear resistance. This also contributes to the increase in the hardness of the coating [36], as was also shown in our study. A maximum hardness from 39.7 GPa to 34 GPa was achieved with a minimum grain diameter of 15.6 nm to 26 nm.

**Figure 12:**
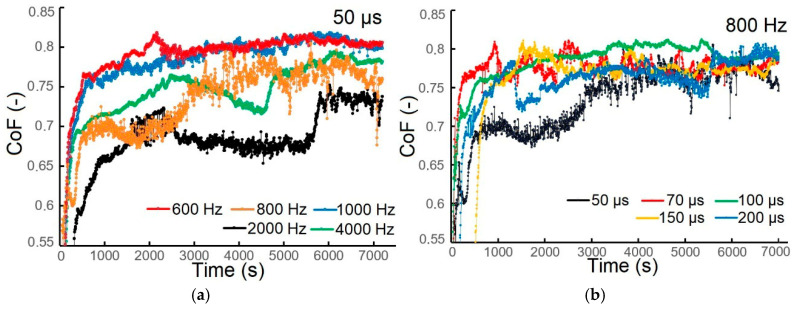
CoF of the TiB_2_ coating as a function of (**a**) frequency at a pulse width of 50 µs and (**b**) pulse width at a frequency of 800 Hz.

The coefficient of friction (CoF) of the TiB_2_ coatings was evaluated using a pin-on-disk test, and dependence on both deposition frequency and pulse width was also analyzed. When the pulse width was kept constant at 50 µs, the CoF decreased as the frequency increased (Figure 12a).

The CoF values for coatings deposited at 600 Hz, 800 Hz, 1000 Hz, 2000 Hz, and 4000 Hz decreased from 0.77 to 0.68. The minimum CoF of 0.68 was measured for the coating deposited at a frequency of 2000 Hz. The CoF increased to 0.72 after approximately 5700 s and remained stable for the rest of the measurement.

**Figure 13:**
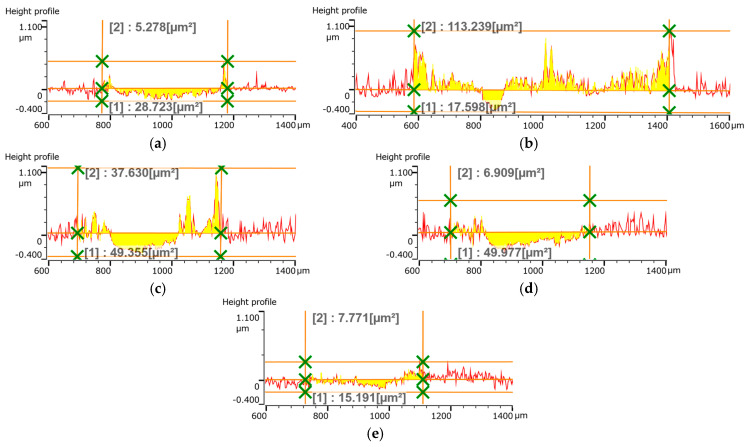
Cross-sectional area of the track after pin-on-disk test of TiB_2_ coatings deposited at a constant pulse width of 50 µs and frequency of (**a**) 600 Hz; (**b**) 800 Hz; (**c**) 1000 Hz; (**d**) 2000 Hz and (**e**) 4000 Hz.

At a constant frequency of 800 Hz, the pulse width had a minimal effect on the CoF, with values consistently close to 0.75 (Figure 13b). The minimum value of CoF was 0.75 for the coating deposited at a pulse width of 50 µs. Firstly, the CoF value increased to 0.67, followed by a decrease and a slight increase to 0.68 until 4000 s. This value remained constant until the end of the measurement, after which it sharply increased up to 0.75.

Wear (loss of TiB_2_ coating material) also corresponded to the tendency associated with hardness and grain size. Cross-sections of the tracks after the pin-on-disk test indicated an adhesive and subsequently abrasive wear mechanism. In the case of a shallow track achieved after the test (constant depth), the hardness of the TiB_2_ coating was high. In the case of an uneven cross-sectional profile of the surface after the test, the coating particles were torn off and subsequently the cutting mechanism occurred.

The COF values obtained during our experiments were higher than those of Deambrosis et al. [47], who reported a CoF of 0.58 for a TiB_2_ coating (1000 Hz, 200 µs pulse width, −50 V bias, 300 °C temperature). The result for this value was 30% lower than in our research study (200 µs, 800 Hz, 300 °C, −60 V bias).

**Figure 14:**
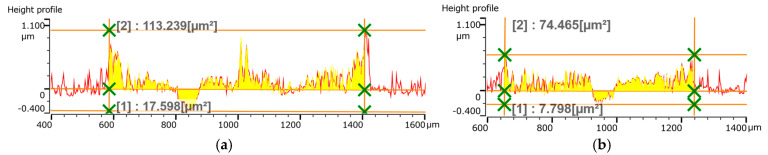

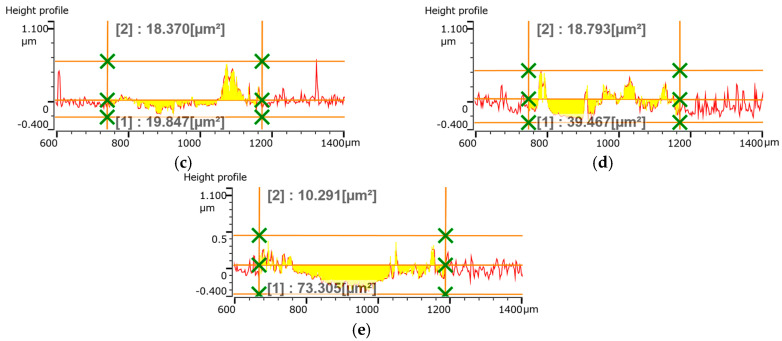
Cross-sectional area of the track after pin-on-disk test of TiB_2_ coatings deposited at a constant frequency of 800 Hz and pulse width of (**a**) 50 µs; (**b**) 70 µs; (**c**) 100 µs; (**d**) 150 µs and (**e**) 200 µs.

In the next stage, the wear was evaluated as the cross-sectional area of the wear track on the pin-on-disk test. Figure 13 and Figure 14 show that the wear track consists of material loss and material pushed to the sides of the path. The pushed-away material can be a combination of the TiB_2_ coating and the sphere’s material, though this was not specifically evaluated.

**Figure 15:**
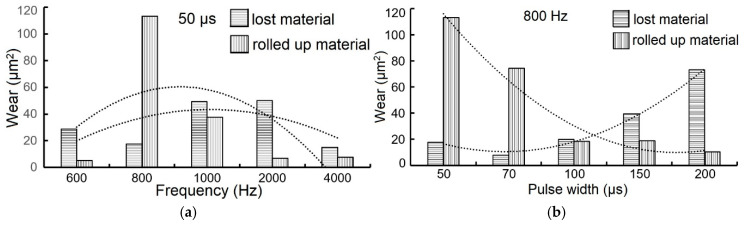
Dependence of wear (cross-sectional area after pin-on-disk test) of TiB_2_ coatings on (**a**) frequency, pulse width 50 µs and (**b**) pulse width at a frequency of 800 Hz.

Figure 15a shows that the minimum material loss occurred at a frequency of 800 Hz, while the maximum loss was at 1000 Hz and 2000 Hz. The amount of pushed-away material showed a similar trend, with a minimum at 800 Hz and a maximum at 600 Hz.

As shown in Figure 15b, increasing the pulse width caused an increase in material loss but a decrease in the amount of material pushed to the sides. This behavior is likely related to the coating’s hardness and the presence of microdroplets, which influence its roughness. The tribological behavior of these coatings warrants further detailed research.

## 4. Conclusions

This study investigated the influence of HiPIMS technological parameters on the properties of TiB_2_ coatings, specifically focusing on frequency and pulse width. The coatings exhibited a single hexagonal TiB_x_ crystal phase with a dominant (001) orientation, confirming the successful deposition of the desired structure. We successfully obtained both stoichiometric (TiB_2_) and overstoichiometric (TiB_x_) compositions, demonstrating the versatility of the HiPIMS method. We observed a clear relationship between the varied parameters and the coating’s properties:

(1) The grain size increased significantly with both increasing frequency (from 15.6 nm to 27.7 nm) and increasing pulse width (from 22.6 nm to 35.4 nm).
(2) Both hardness and Young’s modulus showed a decrease as frequency and pulse width increased. The hardness ranged from approximately 25 GPa to 21 GPa, while Young’s modulus decreased from 450 GPa to 380 GPa. This suggests a strong correlation with the obtained compositions and internal stress.
(3) All coatings demonstrated excellent adhesion, confirmed by Mercedes test ratings of HF1 to HF2. The coefficient of friction (CoF) showed a narrow range of 0.68 to 0.79, indicating stable tribological behavior. Wear, however, decreased with increasing frequency and increased with increasing pulse width, a behavior linked to the balance between hardness and surface roughness.
(4) The coating thickness and roughness (S_a_) increased with increasing frequency and pulse width, confirming that these parameters directly influence the deposition rate and surface morphology.

The quantitative results highlight that the HiPIMS method allows for the precise tuning of TiB_2_ coating properties by adjusting frequency and pulse width. The observed decrease in hardness and Young’s modulus with increasing parameters is likely due to the presence of softer TiB_x_ phases and the influence of higher adatom energy, which can lead to larger grains and a more relaxed coating structure.

The excellent adhesion and stable tribological performance (low CoF value) demonstrate that these coatings are highly suitable for practical industrial applications. The ability to control properties such as grain size, hardness, and wear resistance by simply adjusting deposition parameters provides significant flexibility for tailoring coatings for specific functional requirements, such as those in machining tools or protective coatings where improved longevity is crucial.

This study confirms the potential of the HiPIMS method as a controllable technique for producing high-performance TiB_2_ coatings.

## Figures and Tables

**Table 1:** Chemical composition of TiB_2_ coatings (depending on frequency).

Elemental Composition	Frequency (Hz)—Constant Pulse Width 50 µs				
	600	800	1000	2000	4000
B (at.%)	79.4	66.9	68	68.9	70.8
Ti (at.%)	25.1	33.1	32	31.1	29.2

**Table 2:** Chemical composition of TiB_2_ coatings (depending on pulse width).

Elemental Composition	Pulse Width (Hz)—Constant Frequency 800 Hz				
	50	70	100	150	200
B (at.%)	66.9	74.5	66.2	67.6	78.8
Ti (at.%)	33.1	25.5	33.8	32.4	21.2

## Data Availability

The original contributions presented in this study are included in the article/Supplementary Materials. Further inquiries can be directed to the corresponding authors.

## Supplementary Materials

The following supporting information can be downloaded at https://www.mdpi.com/article/10.3390/ma18204699/s1, Figure S1: Dependence of TiB_2_ coating thickness on: (a) frequency at a pulse width 50 µs and (b) pulse width at a frequency 800 Hz; Figure S2: Roughness parameter value, S_a_ (surface 258 µm × 258 µm and 32 µm × 32 µm) of TiB_2_ coatings deposited at constant pulse with 50 µs and frequency: (a,b) 600 Hz; (c,d) 800 Hz; (e,f) 1000 Hz; (g,h) 2000 Hz and (i,j) 4000 Hz; Figure S3: Roughness parameter value, S_a_ (surface 258 µm × 258 µm and 32 µm × 32 µm) of TiB_2_ coatings deposited at constant frequency 800 Hz and pulse with: (a,b) 50 µs; (c,d) 70 µs; (e,f) 100 µs; (g,h) 150 µs and (i,j) 200 µs; Figure S4: Optical image of adhesion tests results of TiB_2_ coatings deposited at: (a) frequency 800 Hz and pulse length 70 µs, grade HF2; (b) frequency 4000 Hz and pulse length 50 c, grade HF1; Figure S5: XPS spectrum of TiB_2_ coating deposited at a constant pulse width of 50 µs and frequency 600 Hz;Table S1: Crystal size of TiB_2_ coatings depending on pulse frequency; Table S2: Crystal size of TiB2 coatings depending on pulse width
