# Supplementary material for: The Effect of HiPIMS Pulse Conditions on the Microstructural, Mechanical, and Tribological Properties of TiB2 Coatings on Steel Substrates
Source: Materials (Basel). 2025 Oct 13;18(20):4699. doi: 10.3390/ma18204699 (PMC12565820; doi:10.3390/ma18204699)
Supplement: Supplementary file 1 [file materials-18-04699-s001.zip › materials-3874366-supplementary.pdf]

Supplementary Material

The effect of HiPIMS pulse conditions on the microstructure, mechanical, and tribological properties of TiB_2 coatings on steel substrates

Daniel Kottfer[a]*, Karol Kyzioł[b], Mária Kaňuchová[c], Marta Kianicová[a], Michal Žitňan[d], Ewa Durda[b], Marianna Trebuňová[f] , Dávid Medveď[e], Patrik Kľučiar[a]

[a] Faculty of Special Technology, Alexander Dubček University of Trenčín, Ku Kyselke 469, 911 06 Trenčín, Slovakia; daniel.kottfer@tnuni.sk (D.K.), marta.kianicova@tnuni.sk (M.Ki.); patrik.kluciar@tnuni.sk (P.K.)

[b] Faculty of Materials Science and Ceramics, AGH University of Krakow, A. Mickiewicza Av. 30, 30 059, Kraków, Poland; kyziol@agh.edu.pl (K.K.); edurda@agh.edu.pl (E.D.)

[c] Institute of Mountainous Sciences and Environmental Protection, Faculty of Mining, Ecology, Process Control and Geotechnology, Technical University of Košice, Park Komenského 19, 043 84 Košice, Slovakia; maria.kanuchova@tuke.sk (M.Ka.)

[d] Centre for Functional and Surface Functionalized Glass, Alexander Dubček University of Trenčín, Študentská 2, 911 50 Trenčín, Slovakia; michal.zitnan@tnuni.sk (M.Z.)

[e] Institute of Materials Research, Slovak Academy of Sciences, Watsonova 47, 040 01 Košice, Slovakia; dmedved@saske.sk (D.M.)

[f] Institute of special engineering processes, Department of Biomedical Engineering and Measurement, Institute of special engineering processes, Faculty of Mechanical Engineering, Technical University of Košice, Letná 9, 042 00 Košice, Slovakia; marianna.trebunova@tuke.sk (M.T.)

**Correspondence: daniel.kottfer@tnuni.sk*

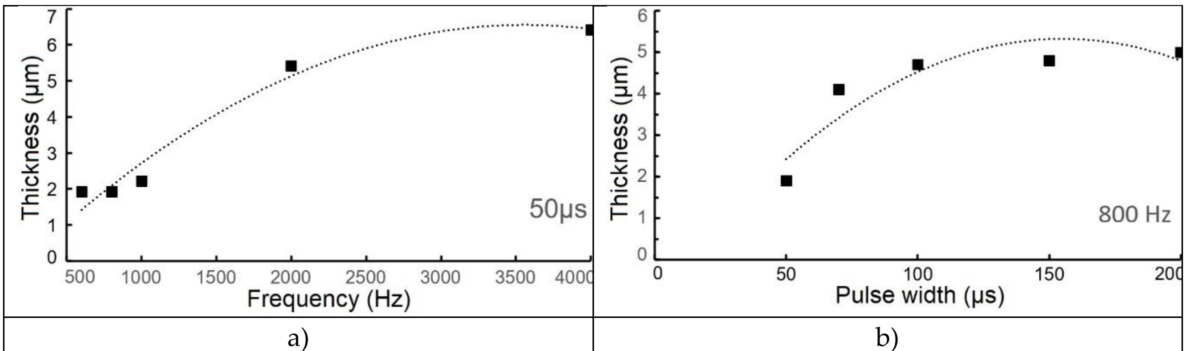

a)	b)

Fig. S1. Dependence of TiB_2 coating thickness on: a) frequency at a pulse width 50 µs and b) pulse width at a frequency 800 Hz.

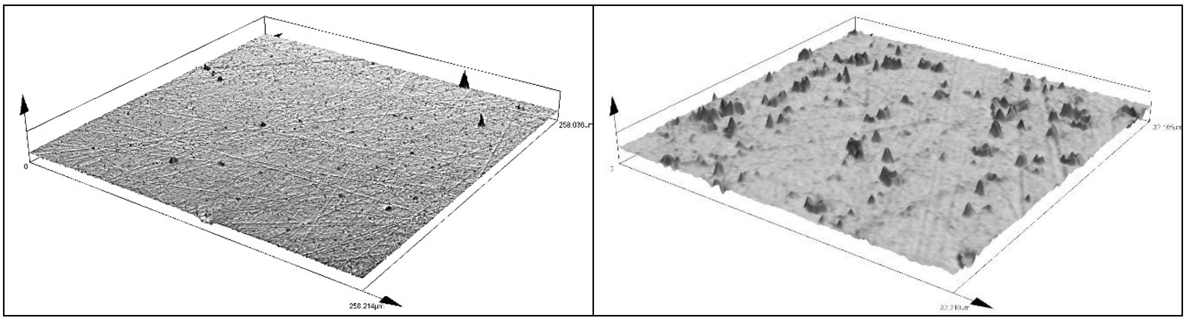

a)	b)
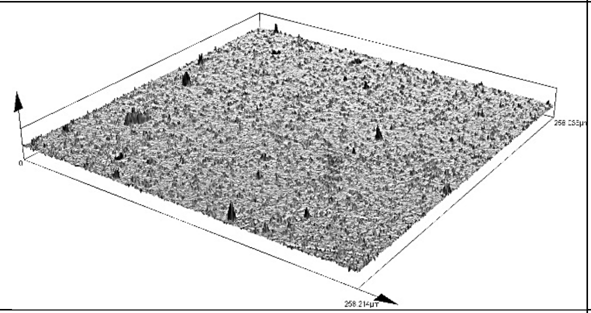	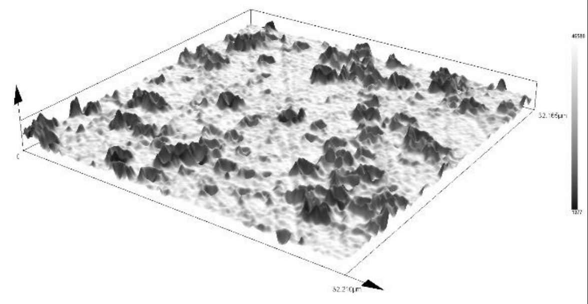
c)	d)
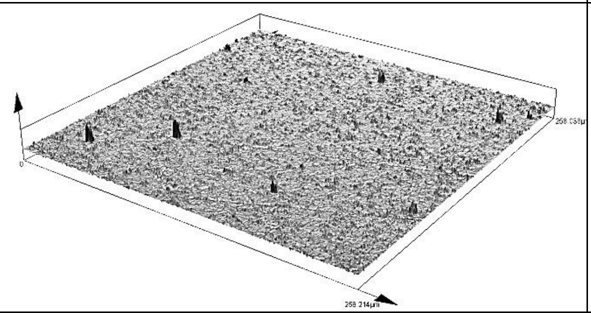	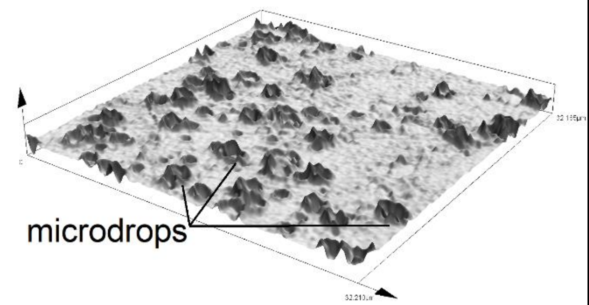
e)	f)
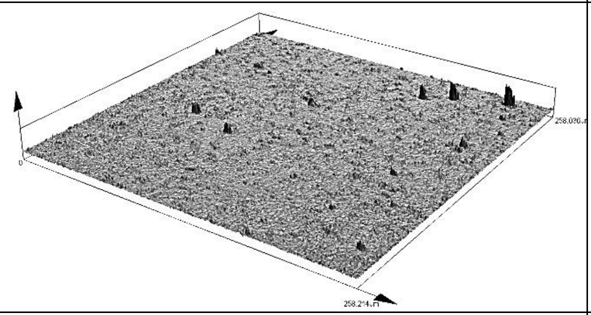	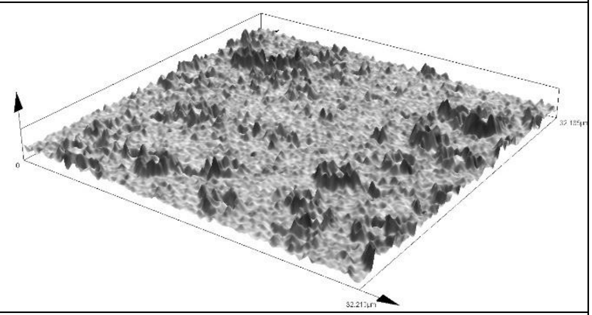
g)	h)
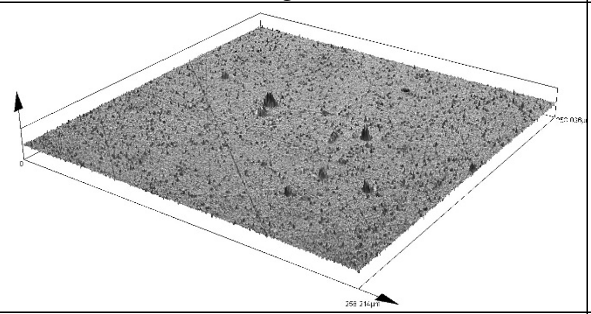	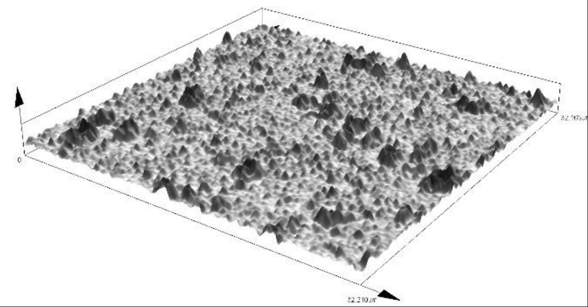
i)	j)

Fig. S2. Roughness parameter value, S_a (surface 258 μm×258 μm and 32 μm×32 μm) of TiB_2 coatings deposited at constant pulse with 50 μs and frequency: a, b) 600 Hz; c, d) 800 Hz; e, f) 1000 Hz; g, h) 2000 Hz and i, j) 4000 Hz

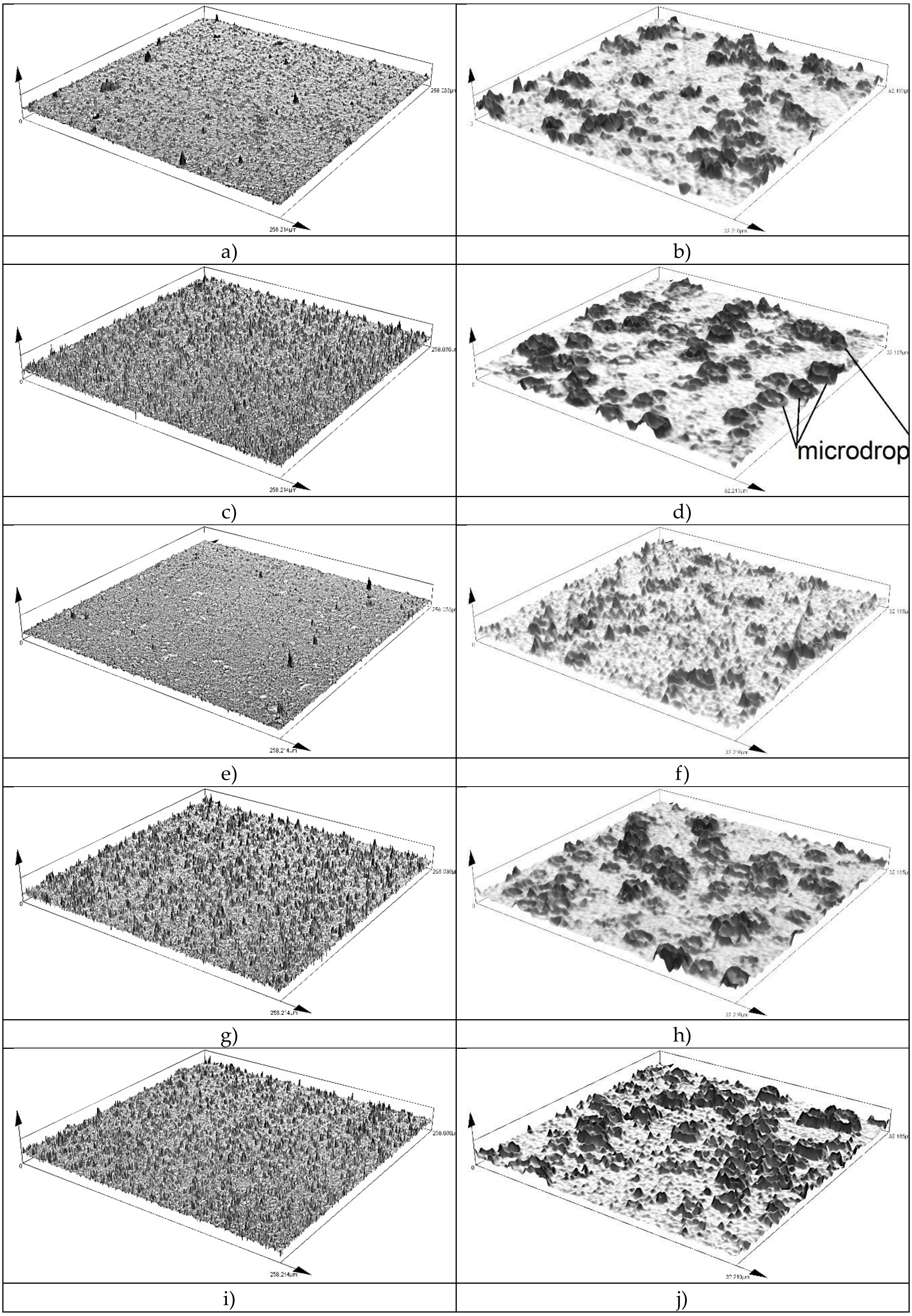

Fig. S3. Roughness parameter value, S_a (surface 258 μm×258 μm and 32 μm×32 μm) of TiB_2 coatings deposited at constant frequency 800 Hz and pulse with: a,b) 50 μs; c, d) 70 μs; e, f) 100 μs; g, h) 150 μs and i, j) 200 μs

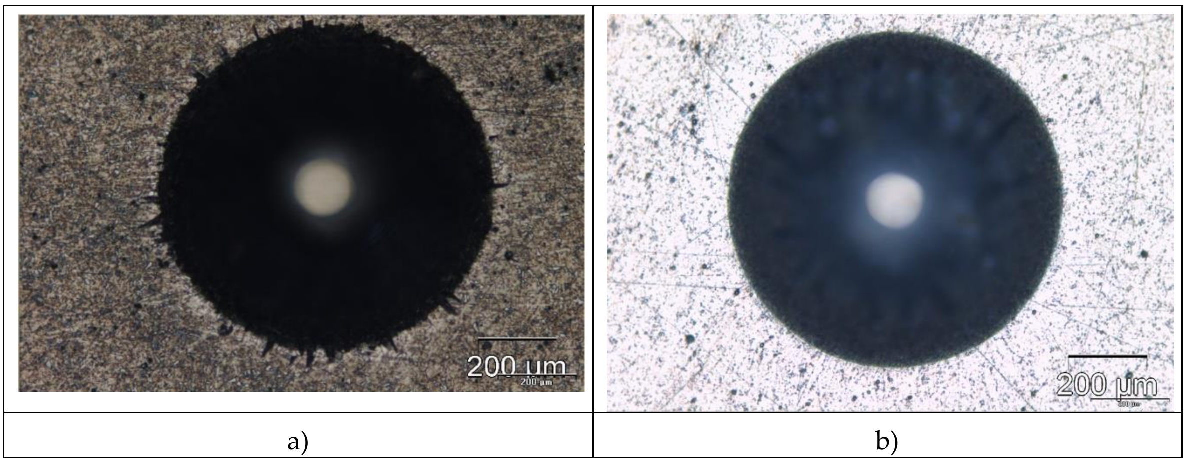

a)	b)

Fig. S4. Optical image of adhesion tests results of TiB_2 coatings deposited at: a) frequency 800 Hz and pulse length 70 µs, grade HF2; b) frequency 4000 Hz and pulse length 50 c, grade HF1.

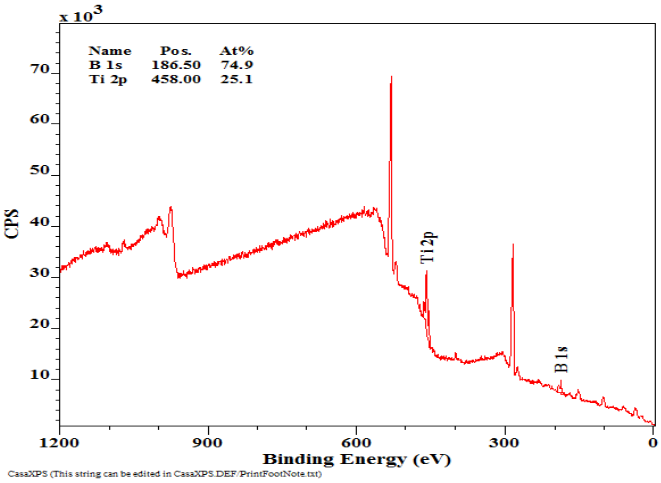

Fig. S5. XPS spectrum of TiB_2 coating deposited at a constant pulse width of 50 µs and frequency 600 Hz

Table S1. Crystal size of TiB_2 coatings depending on pulse frequency

Frequency [Hz]	Crystal size [nm]
600	15.6
800	22.6
1000	26.1
2000	13.6
4000	27.7

Table S2. Crystal size of TiB_2 coatings depending on pulse width

Pulse width [µs]	Crystal size [nm]
50	22.6
70	29.0
100	26.7
150	35.4
200	35.2
